# Santonine Poisoning

**Published:** 1878-05

**Authors:** 


SANTONINE POISONING.—Prof. F. Forchheimer (*The Clinic*, January 5th, 1878) summaries the symptoms induced by a toxic dose of santonine as follows: "Sopor, the eyes fixed, pupils dilated and without reaction, the lips red and swollen, the respiration stertorious, pulse slow, skin cold. These symptoms become more and more intense, and suddenly convulsions set in, which rapidly extend to the muscles of respiration, so that respiration becomes very slow. Irregular and long pauses occur, in which the breathing seems to be entirely stopped. With all this the patient usually passes urine involuntarily drop by drop."

Chloral hydrate is antagonistic to santonine, and may be used carefully. Inhalation of ether controls the spasms.

